# A nondestructive method of calculating the wing area of insects

**DOI:** 10.1002/ece3.8792

**Published:** 2022-04-01

**Authors:** Kexin Yu, Gadi V. P. Reddy, Julian Schrader, Xuchen Guo, Yirong Li, Yabing Jiao, Peijian Shi

**Affiliations:** ^1^ 74584 College of Biology and the Environment Bamboo Research Institute Nanjing Forestry University Nanjing China; ^2^ USDA‐ARS‐Southern Insect Management Research Unit Stoneville Mississippi USA; ^3^ School of Natural Sciences Macquarie University Sydney New South Wales Australia; ^4^ Biodiversity, Macroecology and Biogeography University of Göttingen Göttingen Germany; ^5^ Tropical Silviculture and Forest Ecology University of Göttingen Göttingen Germany

**Keywords:** forewings, proportional relationship, scaling, wing length, wing width

## Abstract

Most insects engage in winged flight. Wing loading, that is, the ratio of body mass to total wing area, has been demonstrated to reflect flight maneuverability. High maneuverability is an important survival trait, allowing insects to escape natural enemies and to compete for mates. In some ecological field experiments, there is a need to calculate the wing area of insects without killing them. However, fast, nondestructive estimation of wing area for insects is not available based on past work. The Montgomery equation (ME), which assumes a proportional relationship between leaf area and the product of leaf length and width, is frequently used to calculate leaf area of plants, in crops with entire linear, lanceolate leaves. Recently, the ME was proved to apply to leaves with more complex shapes from plants that do not have any needle leaves. Given that the wings of insects are similar in shape to broad leaves, we tested the validity of the ME approach in calculating the wing area of insects using three species of cicadas common in eastern China. We compared the actual area of the cicadas’ wings with the estimates provided by six potential models used for wing area calculation, and we found that the ME performed best, based on the trade‐off between model structure and goodness of fit. At the species level, the estimates for the proportionality coefficients of ME for three cicada species were 0.686, 0.693, and 0.715, respectively. There was a significant difference in the proportionality coefficients between any two species. Our method provides a simple and powerful approach for the nondestructive estimation of insect wing area, which is also valuable in quantifying wing morphological features of insects. The present study provides a nondestructive approach to estimating the wing area of insects, allowing them to be used in mark and recapture experiments.

## Graphical abstract

Forewing area was found to be proportional to the product of forewing length and width for each of three cicada species, which followed the Montgomery equation. The mixed dataset of the three cicada species also followed the Montgomery equation, which suggests that this equation can apply to the calculation of wing area for other insects with similar wing shapes.

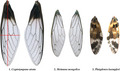

## 1 INTRODUCTION

The wing morphology of insects strongly affects aerodynamic performance, and the diverse array of wing shapes reflects the insects’ adaptation to environmental differences (Aiello et al., 2021; Ellington, 1984). Wing loading (defined as body mass per unit wing area) has been demonstrated to reflect flight maneuverability of insects to a large extent (Byrne et al., 1988; Grabow & Rüppell, 1995; Mena et al., 2020), so calculation of wing area is of value in flight performance studies with insects. However, wing morphological diversity makes it difficult to develop a general geometric equation to describe wing outlines of insects. Some methods were proposed by previous studies (Byrne et al., 1988; Ellington, 1984; Fischbein et al., 2018; Gyulavári et al., 2017) to estimate the wing area of insects: (i) photographing wings of insects and marking a number of landmarks on the wing's boundary, which form a polygon, to calculate the wing area; (ii) scanning wings of insects by photo scanners and using image processing software (e.g., ImageJ) to calculate the wing area based on scanned images; (iii) developing parametric models to calculate wings of insects via the integral. The first method can lead to large measure errors due to the photographing distance and angle, and resulting from the limitation of the number of landmarks sampled on the wing's boundary. The second method is destructive as the wings need to be detached from the body. The third method also requires the detachment of wings from the body, and is only suitable for special wing shapes (Ellington, 1984) rather than serving as a general method for different wing shapes. Overall, the previous methods of calculating the wing area of insects are time consuming and destructive, which indicates the need for developing fast and nondestructive methods to measure the wing area of insects.

This problem is analogous to measuring leaf area in plant functional ecology. The leaf area is an important functional trait linked to plant growth strategy and environmental adaptations (Baird et al., 2021; Wright et al., 2017). Plant leaves can have many different shapes, such as elliptical, lanceolate, linear, oblong, ovate, palmate, pinnate, and so on (Ellis et al., 2009; Runions et al., 2017), making leaf‐area estimation complicated. Fortunately, much progress has been made in recent years to accurately estimate leaf area based on simple, allometric relationships that also work nondestructively (Schrader et al., 2017, 2021; Shi, Liu, Ratkowsky, et al., 2019; Shi, Liu, Yu, et al., 2019; Yu et al., 2020). Especially promising approaches are allometric relationships of leaf dimensions, such as length and width, with leaf area (Shi, Liu, Ratkowsky, et al., 2019), which are likely to be extended to the area estimation of other biological organs, for example, insect wings.

Allometic relationships for leaf area estimation are based on the principle of similitude proposed by Thompson (1917), which assumed that the area of an object was proportional to the square of its length. Accordingly, the leaf area and length tend to have a power‐law relationship with a scaling exponent of 2 (Shi, Miao, et al., 2022). However, the accuracy of the use of the principle of similitude significantly depends on the extent of the variation in the ratio of leaf width to length (Shi et al., 2021; Shi, Liu, Ratkowsky, et al., 2019; Yu et al., 2020). If the conspecific variation in this ratio is large, the principle of similitude does not hold true, leading to a large prediction error using the square of leaf length to calculate leaf area. Montgomery (1911) assumed that leaf area of corn is proportional to the product of leaf length and width, which is referred to as Montgomery equation (ME) for convenience hereinafter. The validity of ME applies to many complex, flat‐leaf shapes, and most estimates of the proportionality coefficient (i.e., the Montgomery parameter, MP) in ME were found to range from 1/2 to *π*/4 (Schrader et al., 2021; Shi et al., 2021; Shi, Liu, Ratkowsky, et al., 2019; Shi, Liu, Yu, et al., 2019; Yu et al., 2020). The forewings of many insects exhibit a subelliptical shape but do not follow a classical geometric shape, so it is impossible to measure their areas based on a known parametric equation accurately. However, the forewing shapes of insects are less complex than some extreme leaf shapes (e.g., lobed or serrated leaves; see Schrader et al. [2021] for examples). Thus, we hypothesize that ME can also be applied to the calculation on the forewing area of insects.

Cicadas as a conspicuous group of insects occur in both temperate and tropical regions and are a highly diverse group with over 3390 species (Sanborn, 2014; Williams & Simon, 1995). Due to their relatively large body size (compared to other insects), cicadas are popular study animals for research on flight mechanisms, which could lead to aerodynamics designs for flapping, wing‐based micro air vehicles (Ellington, 1999; Wan et al., 2015). However, the macroscopic features of wings (including wing size and shape) of cicadas have attracted less attention. It is unknown whether wing shape varies principally with wing size as it increases across closely related species within the same taxon. Here, we studied the wings of three cicada species (in three different genera) that are commonly found in eastern China. We first tested whether ME can be used to describe a proportional relationship between forewing area and the product between forewing length and width. Second, we compared whether differences existed between pairs of species in the estimates of MP. Third, we pooled the data for the three cicada species and further examined whether ME is still valid in calculating wing area for the mixed dataset.

## 2 MATERIALS AND METHODS

### 2.1 Wing sampling

**FIGURE 1:**
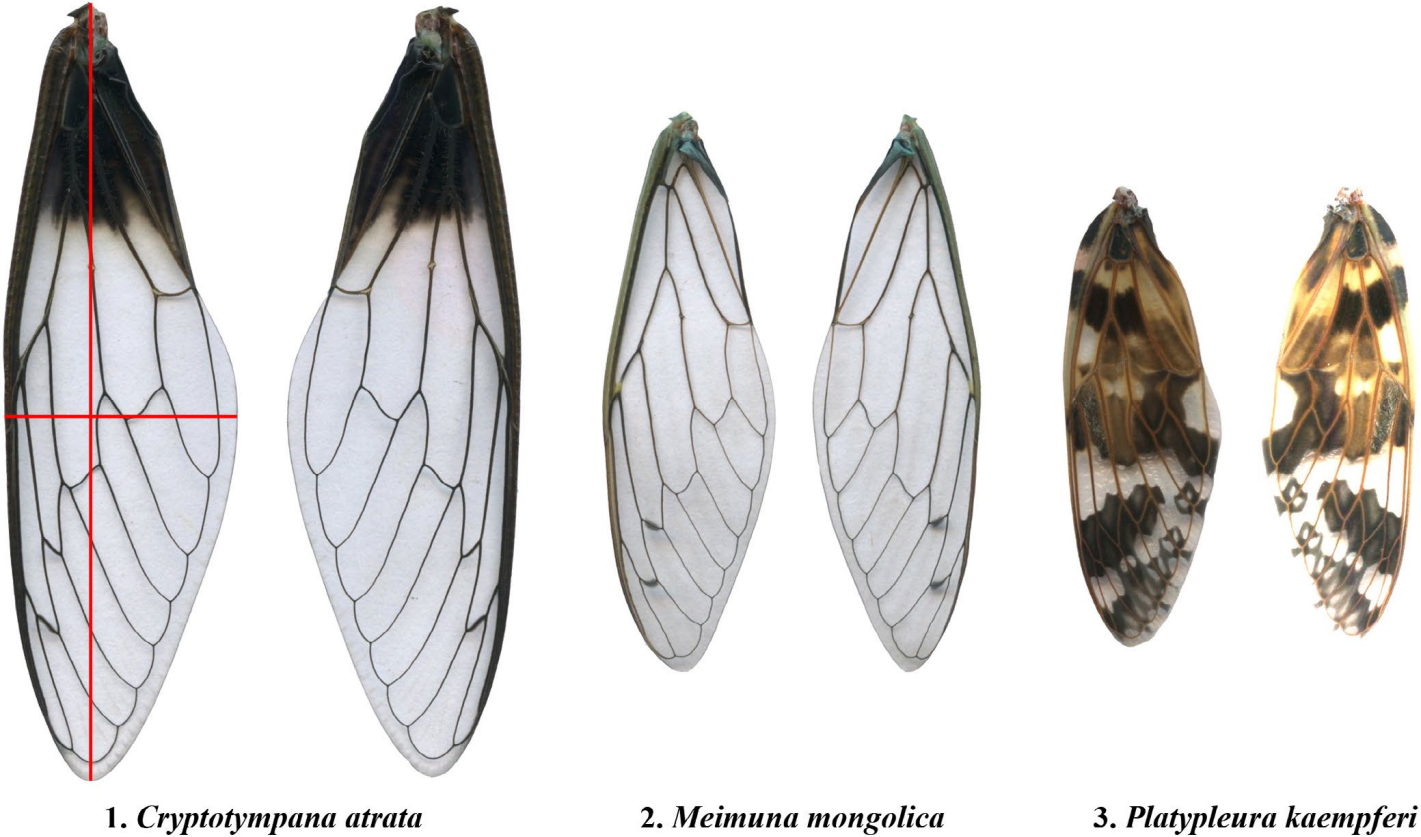
Forewing examples for the three cicada species studied. The red horizontal and vertical lines represent the forewing width and length

We caught 395 individuals of three cicada species (24 of *Cryptotympana atrata* [Ca], 243 of *Meimuna mongolica* [Mm], and 128 of *Platypleura kaempferi* [Pk]) at the Nanjing Forestry University campus (32°4'41''N, 118°48'34''E) from 6 to 25 July 2021. Cicada sampling was carried out at night (19:40–21:40). We collected nymphs emerging from the soil and adults that had just shed their nymphal exoskeletons and were drying their wings on trunks or branches. Captured insects were held at ca. 25°C for one night to allow the wings of all the cicadas to ultimately expand. The sex of each cicada was recorded. We can directly photograph wings from living insects using a camera with a scale ruler, and this does not affect the extraction of the data points on the wing boundaries (Li, Quinn, Gielis, et al., 2022). Nevertheless, we needed to analyze the scaling relationship between total wing area and body mass in another study (Shi, Jiao, et al., 2022). It was inconvenient to measure body mass of living insects with wings. In that case, we detached the forewings with a scalpel and directly scanned detached wings. Because the sample size of Ca (24) from the Nanjing Forestry campus was too small, we collected more Ca adults that were resting on trees using a metal rod tipped with sticky flour, from 15 to 26 August 2021 at another site (32°6'25''N, 118°57'3''E; nearby Nanjing University Xianlin campus) 13.7 km away from the Nanjing Forestry University campus. See Table 1 for sampling details. We did not consider the difference in wing outlines between males and females for the same species because there were no significant differences. If ME can apply to the pooled data of the two sexes, it surely does so for males or females separately. We only used forewings as study materials because forewing shapes of cicadas are similar to those of a variety of insects (Figure 1).

**TABLE 1:** Sampling information for the three cicada species used in this study

Species code	Latin name	Sampling site	Sample size		
			Male	Female	Total
Ca	*Cryptotympana atrata*	NFU, NUX	76	88	164
Mm	*Meimuna mongolica*	NFU	234	252	486
Pk	*Platypleura kaempferi*	NFU	86	170	256

Note

NFU represents Nanjing Forestry University campus; NUX represents Nanjing University Xianlin campus.

### 2.2 Image processing

Cicada forewings were scanned at a resolution of 600 dpi using an Epson photo scanner (Epson V550, Batam, Indonesia). Then, the scanned images were converted into black‐white images and saved as bitmap images at the resolution of 600 dpi using Adobe Photoshop CS2 (version 9.0; Adobe, San Jose, CA, USA; https://www.adobe.com/products/photoshop.html). We used the M‐file of MATLAB (version 2009a; MathWorks, Natick, MA, USA; https://www.mathworks.com/products/matlab.html) developed by Shi et al. (2018) to extract the planar coordinates of the wing outlines. The script based on R (version 3.6.1; R Core Team, 2019) developed by Su et al. (2019) was used to calculate wing area, length, and width. We defined the maximum distance from the wing base to wing tip as wing length, and the maximum distance of any two points on the wing's boundary perpendicular to the wing length axis as maximum wing width. Figure 1 shows the wing shapes of the three cicada species.

All the raw data can be found in Dryad, a public repository (see Shi, 2022).

### 2.3 Data analysis

We compared six models as candidates for calculating wing area (Table 2). Model 1 (i.e., ME) assumes that the wing area is proportional to the product of wing length and width; model 2 assumes that the wing area has a power‐law relationship with the product of wing length and width; models 3 and 5 assume that the wing area is proportional to the square of wing length, and that of wing width, respectively; models 4 and 6 assume that the wing area has a power‐law relationship with wing length and wing width, respectively. Model 2 considers the influence of a deviation between the planar projection and the actual surface of a wing on the calculation of wing area, and it suggests a power‐law relationship between these two variables to reflect such an influence, since the wing surface is not entirely flat but an expansion in 3D space. If the wing surface is sufficiently flat, the scaling exponent of wing area versus the wing length and width product will approach 1. Two sides of each model were transformed into logarithmic forms to stabilize the variance of the observations of wing area for data fitting (Table 2), and then data were fitted by using the ordinary least‐squares method. For models 2, 4, and 6, we calculated the 95% confidence intervals of the slope using the bootstrap percentile method (Efron & Tibshirani, 1993; Sandhu et al., 2011). The bootstrap percentile method was also used to test whether there was a significant difference in the estimates of MP (i.e., *c*_1_ of model 1 in Table 2) between any two species. For each dataset corresponding to one species, 4000 bootstrap replicates of MP were obtained from fitting bootstrap samples. We checked whether the 95% confidence interval (CI) of the differences between any two groups of bootstrap replicates of MP included 0. If it includes 0, there is no significant difference between the two estimates of MP; if it does not include 0, there is a significant difference between those estimates. If the upper bound of the 95% CI of MP differences is smaller than 0, it indicates that the first estimate of MP is significantly smaller than the second one; if the lower bound of the 95% CI of MP differences is greater than 0, it indicates that the first estimate of MP is significantly larger than the second one. See Sandhu et al. (2011) for details.

**TABLE 2:** A description of the six models used in this study

Model no.	Model	Log‐transformed model
Model 1	$A=c_{1}\left(\mathrm{LW}\right)$	$\ln\left(A\right)=a_{1}+\ln\left(\mathrm{LW}\right)$
Model 2	$A=c_{2}{\left(\mathrm{LW}\right)}^{b_{2}}$	$\ln\left(A\right)=a_{2}+b_{2}\ln\left(\mathrm{LW}\right)$
Model 3	$A=c_{3}L^{2}$	$\ln\left(A\right)=a_{3}+2\ln\left(L\right)$
Model 4	$A=c_{4}L^{b_{4}}$	$\ln\left(A\right)=a_{4}+b_{4}\ln\left(L\right)$
Model 5	$A=c_{5}W^{2}$	$\ln\left(A\right)=a_{5}+2\ln\left(W\right)$
Model 6	$A=c_{6}W^{b_{6}}$	$\ln\left(A\right)=a_{6}+b_{6}\ln\left(W\right)$

Note

Here, *c* is equal to exp $\hat{a}$; the subscripts represent the different models; *A* represents wing area; *L* represents wing length; *W* represents wing width. In log‐transformed models, *a* represents the intercept; *b* represents the slope; the subscript represents the model no.; the slopes of models 3 and 5 are fixed to be 2 rather than constants to be estimated.

We used the root‐mean‐square error (RMSE) to reflect the goodness of fit of the six models, where RMSE was defined as Equation 1

(1) $$\text{RMSE}=\sqrt{\sum_{i=1}^{n}{\left(y_{i}‐{\hat{y}}_{i}\right)}^{2}/n},$$

where *y* represents the natural logarithm of the observed wing area; *i* represents the *i*th wing; and *ŷ* is the value predicted by a model. The smaller the RMSE value is, the better the goodness of fit is. We used the absolute percentage error (APE, see Equation 2) to measure the discrepancy between the *j*th RMSE value and the *i*th RMSE value to determine whether it is worth adding an additional parameter in a model (He et al., 2020; Yu et al., 2020):

(2) $${\text{APE}}_{\mathrm{ij}}=\left|\frac{{\text{RMSE}}_{i}‐{\text{RMSE}}_{j}}{{\text{RMSE}}_{i}}\right|\times 100\%.$$

When two models have approximate structures but one has an additional parameter relative to another (e.g., model 1 vs. model 2 in Table 2), APE is used to determine whether it is worth increasing an additional parameter in a model to decrease the prediction error. There is no absolute rule for defining an APE value to choose a better model. As a rule of thumb, if APE > 5%, we usually accept a model with *n* + 1 parameters rather than the model with *n* parameter; if APE ≤ 5%, we usually reject the model with *n* + 1 parameters. In our analyses, the APE values of model 1 versus 2, model 3 versus 4, and model 5 versus 6 were compared, respectively.

In addition, we used Tukey's Honestly Significant Difference (HSD) test with a 0.05 significance level (Hsu, 1996) to examine whether there were significant differences in wing area, length, and width between any two species and between males and females of the same species.

## 3 RESULTS

**FIGURE 2:**
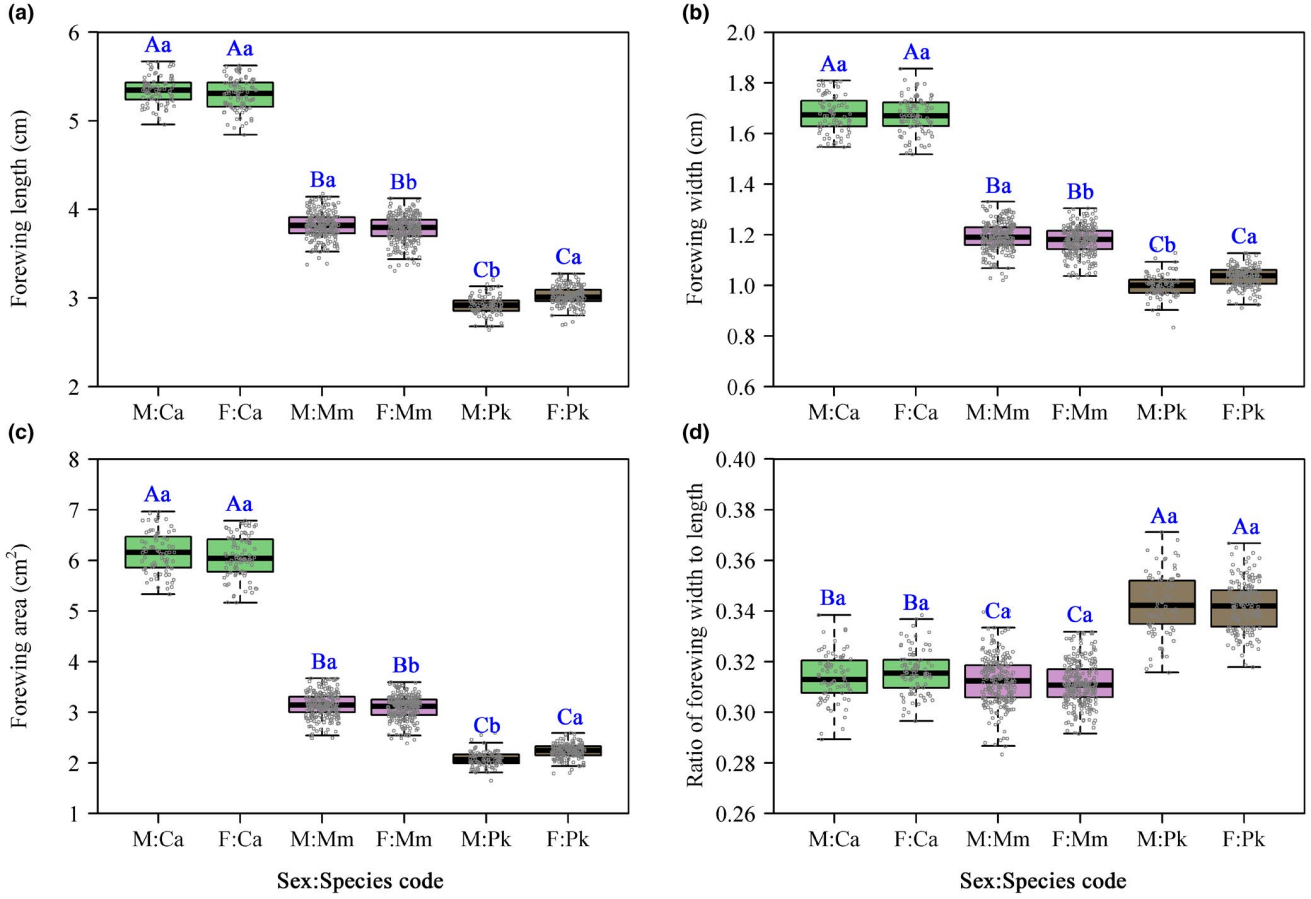
Comparison of forewing length (a), forewing width (b), forewing area (c), and the ratio of forewing width to length (d). The letters at the top of the whiskers represent the significance of the difference between any two species based on the significance level 0.05. The capital letters are for the interspecific comparison, and the lowercase letters for the comparison of two genders. There is no significant difference between any two species (or two sexes) sharing the same letter, and there is a significant difference between any two species (or two sexes) that do not share the same letter. Different colorful boxes represent different species. The species codes are the same as those listed in Table 1

Forewing length, width, and area were significantly different among the three cicada species (Figure 2a–c; for the three species of male cicadas, *F*_2, 391_ = 5994, 2983, and 5181 with all *p* values <.001 corresponding to forewing length, width, and area; for the three species of female cicadas, *F*_2, 509_ = 7335, 4306, and 6820 with all *p* values <.001corresponding to forewing length, width, and area). Ca had the largest forewing size, and Pk had the smallest. There was no significant difference in forewing size between the sexes of Ca (*F*_1, 162_ = 3.35, 0.123, and 1.44 with *p* = .07, .73, and .23 corresponding to forewing length, width, and area), while there was a significant difference in forewing size between the sexes for each of the other two species. For Mm, males had a larger mean forewing size than females (*F*_1, 484_ = 10.03, 11.11, and 8.08 with *p* = .0016, .0009, and .0047 corresponding to forewing length, width, and area); however, for PK, males had a smaller mean forewing size than females (*F*_1, 254_ = 63.32, 37.10, and 58.23 with all *p* values <.001 corresponding to forewing length, width, and area). There was no significant difference in forewing shape (reflected by the ratio of forewing width to length) between sexes for the three species (*F*_1, 162_ = 2.35 with *p* = .127 for Ca; *F*_1, 484_ = 1.15 with *p* = .285 for Mm; *F*_1, 254_ = 0.34 with *p* = .559 for Pk). We found that Pk has the broadest forewing shape and Mm has the narrowest forewing shape (Figure 2d). These differences indicate that the order of the differences for wing shape was not the same as that of wing size (Figure 2c vs. d).

**FIGURE 3:**
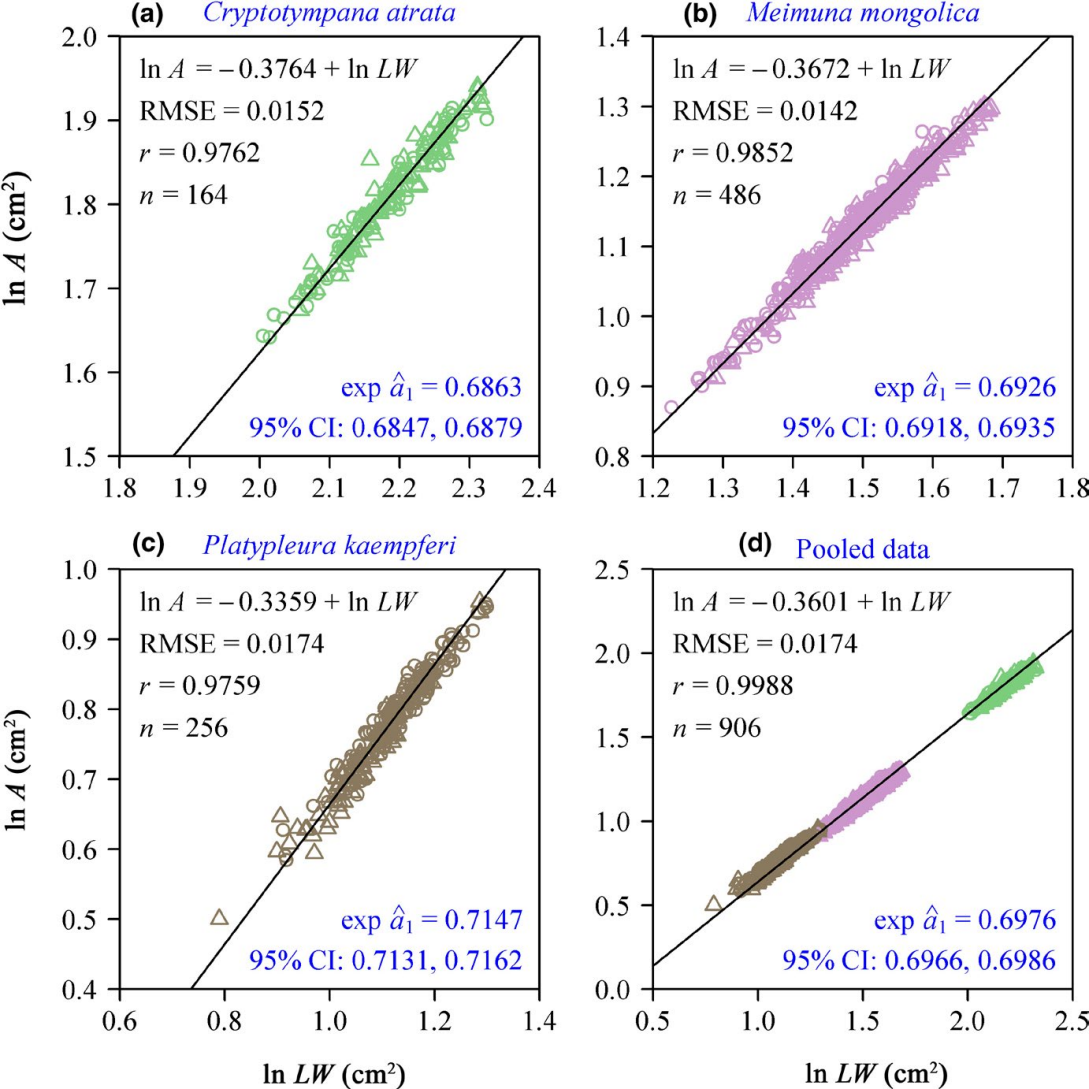
Fitted results using the Montgomery equation for the data of forewing area vs. the product of forewing length and width on a log–log scale. Panels a to c correspond to the results at the species level, and panel d corresponds to the result for the pooled data of the three cicadas. In each panel, RMSE is the root‐mean‐square error of the linear fitting; *r* is the correlation coefficient; *n* is the sample size; exp ${\hat{a}}_{1}$ is the estimated Montgomery parameter; 95% CI is the 95% confidence interval of the estimated Montgomery parameter

**FIGURE 4:**
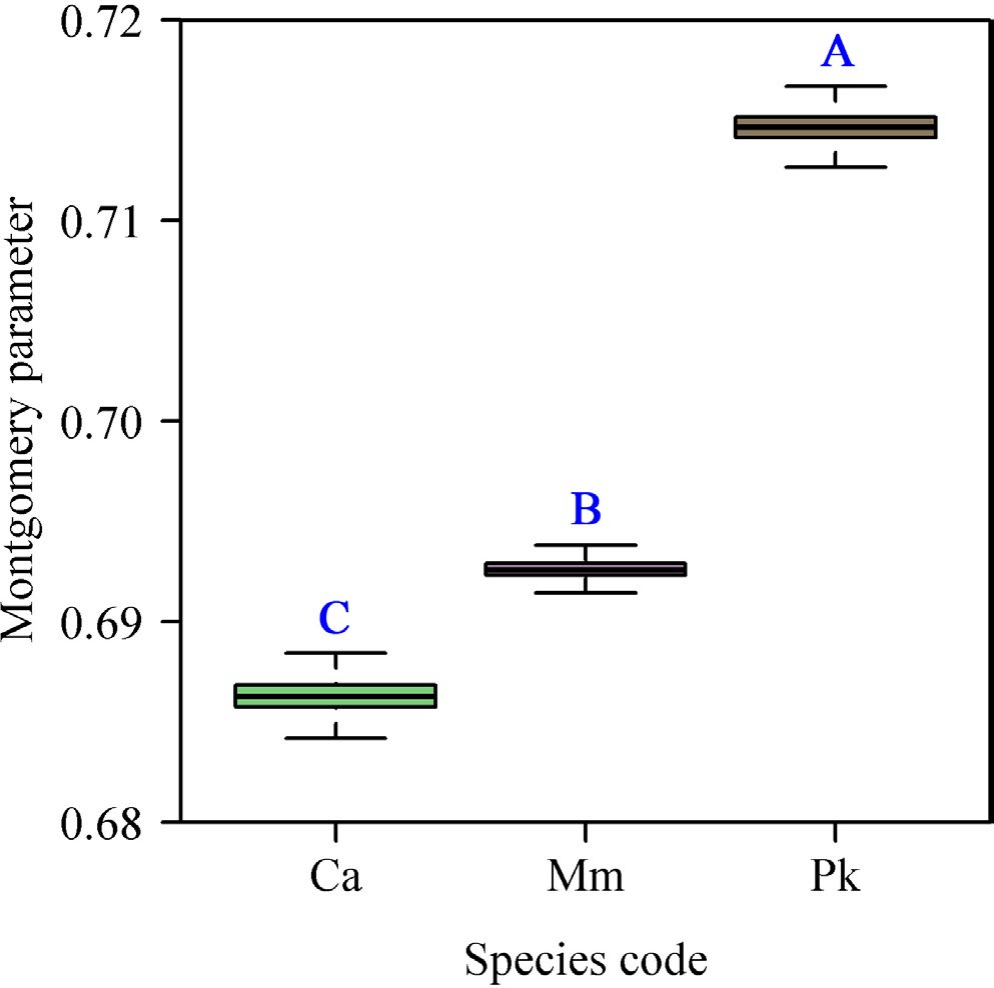
Comparison of the estimates of the Montgomery parameter (MP) among three cicada species. The boxplot came from 4000 bootstrap replicates of MP. Letters A, B, and C are marks of significant differences which signify that the estimated MP of Ca is the smallest and that of Pk is the largest. Take letter A for example. Letter A denotes that the upper bound of the 95% confidence interval (CI) of the 4000 differences in the bootstrap replicates of MP between Mm and Pk is smaller than 0, which suggests that the estimated MP of Mm is significantly smaller than that of Pk

Among the six models, models 1 and 2 had smaller RMSE values (both <0.02) compared with the remaining four models, confirming the efficacy of those two models in calculating the forewing area (Table 3). Although the RMSE values of model 1 were slightly greater than those of model 2, the APE values were between 4.03 and 5.12% (Table 3). From the viewpoint of a trade‐off between the complexity of model structure and the goodness of fit, model 1 (i.e., the Montgomery equation, ME) was considered best. The numerical values of the Montgomery parameters (MPs) for the three cicada species were 0.6863, 0.6926, and 0.7147, respectively (Figure 3). There were significant differences in the numerical values of the MP among the three species (Figure 4). For the pooled data of all three species, the numerical value of MP was 0.6976.

**TABLE 3:** RMSE and APE values between the observation and the predicted value of wing area using the six models and APE values

Species code	RMSE_1_	RMSE_2_	RMSE_3_	RMSE_4_	RMSE_5_	RMSE_6_	APE_12_	APE_34_	APE_56_
Ca	0.0152	0.0146	0.0295	0.0292	0.0357	0.0268	4.03%	0.98%	24.83%
Mm	0.0142	0.0136	0.0316	0.0310	0.0324	0.0260	4.36%	2.10%	19.68%
Pk	0.0174	0.0165	0.0337	0.0319	0.0365	0.0300	5.12%	5.36%	17.70%

Note

Species codes are the same as those in Table 1; RMSE represents the root‐mean‐square errors of a model; the subscripts 1–6 of RMSE represent models 1–6, respectively; APE_12_ represents the absolute percent error between RMSE_1_ and RMSE_2_, and so on.

## 4 DISCUSSION

### 4.1 Validity of ME in calculating wing area

We fitted the data of forewing area versus the product of forewing length and width for each cicada species, and found that the goodness‐of‐fit requirement was met, which means that the Montgomery equation (ME) method was suitable for calculating wing area of subelliptical wing shapes. Previous studies showed that ME is valid for calculating leaf area, both at the species level and at higher taxonomic (e.g., genus, family) levels; for plants, most estimates of MP ranged from 1/2 to *π*/4 (Schrader et al., 2021; Shi et al., 2021; Shi, Liu, Ratkowsky, et al., 2019; Shi, Liu, Yu, et al., 2019; Yu et al., 2020). In our study on cicada wings, MP values at the species level and for the pooled data were both approximately 0.7, which corresponds to the MP values for unlobed ovate, lanceolate, and linear leaves of plants (Schrader et al., 2021; Shi et al., 2021). For both individual species data and pooled data, the correlations between wing area and the product of wing length and width on a log–log scale exceeded 0.975, and the RMSEs of all linear regressions were all smaller than 0.02 (Figure 3). These findings show that ME is also valid for use at the genus or family level if wing shapes among species are similar. Nevertheless, the prediction error may increase when calculating wing areas of multiple species within a higher taxon that may have a larger variation in wing shape. Given that the ME can be successfully applied to leaves with shapes that are more complex than the shapes of insect wings, we argue that ME can be extended to other insects regardless of wing types (e.g., membranous wings or tegmina). However, over evolutionary time, wing types of insects have become more complex. The elytra of beetles are hardened and serve as a protective layer. Therefore, the forewings have a curved surface, with a greater curvature than other groups of insects like butterflies (Sun & Bhushan, 2012). Also, the hind wings of some Diptera insects are reduced and highly modified into small, club‐shaped 3D halteres that help flies remain stable in flight (Agrawal et al., 2017). These wings are not perfectly flat, which might limit the use of ME to a degree, because it is not easy to measure the length and width of the planar projections of wings.

The ME was first used to calculate the area of linear and lanceolate leaf shapes (Montgomery, 1911), and was then found to be applicable of calculating the area of more complex leaf shapes (Schrader et al., 2021; Shi, Liu, Ratkowsky, et al., 2019; Shi, Liu, Yu, et al., 2019; Yu et al., 2020). Its validity relies on the extent of the similarity of studied polygons to a great degree, but it does not depend on the size of studied polygons (Baird et al., 2021; Shi et al., 2021). Recent studies on the cross‐section area of plants, the planar projection area of ginkgo seed and the stomatal area further confirmed this statement (Huang et al., 2020; Tian et al., 2020; Xiong & Flexas, 2020). For insects, the conspecific variation in wing shape is usually smaller than that in wing size. Even for the wing shape across the closely related species within the same taxon, for example, the studied three cicada species here, the variation in wing shape is still small. This ensures the validity of ME. In addition to the leaf shape of broad‐leaved plants and wing shape of insects, it is also a promising method for calculating other morphological measures such as the body projection area of insects when studying the scaling relationship between body size, being represented by body projection area, and weight. At the cell level, the ME can be used to calculate the cell size quickly when the cells are similar in shape, for example, stoma (Xiong & Flexas, 2020). Overall, the ME can be widely used to fast and nondestructively calculate the area of some objects with similar morphological characteristics in botany, ecology, and entomology.

### 4.2 Numerical value of MP and its application in quantifying the complexity of wing shape

Insects are the most diverse and numerous class in the animal kingdom (Misof et al., 2014). Different habitats and climates have resulted in the evolution of diverse wing forms in the insects (Salcedo & Socha, 2020), suggesting that a representative but straightforward parameter to describe wing forms of insects would be valuable. Our study showed that the forewing area of cicadas can be accurately estimated by the product of the wing length and wing width, multiplied by a correction factor (i.e., MP). In addition to the calculation of wing area, the estimated MP can be potentially used as a reference indicator for assisting in species taxonomic identification because, at the species level, significant conspecific differences can be detected (Figure 4). Linnaeus (1758) put forward a classification of insects in his *Systema Naturae* that depended on the nature of their wings (existence, texture, shape, and so on), and he divided insects into Coleoptera, Hemiptera, Lepidoptera, Neuroptera, Hymenoptera, Diptera, and Aptera (Engel & Kristensen, 2013). Traditional insect taxonomy still makes use of the subjective description of wing forms, but such an approach can be somewhat misleading. However, MP values might be a valuable addition to the descriptions of insect wings. Whether MP values can be used as a parameter to distinguish different species deserves further investigation.

Shi, Liu, Ratkowsky, et al. (2019) and Shi, Liu, Yu, et al. (2019) reported that MP values of broad‐leaved plants usually ranged from 1/2 to *π*/4, with the smallest MP value found being for *Polygonum perfoliatum*, which has approximately triangular leaves and an MP value only slightly larger than 0.5. The largest MP value described in Shi, Liu, Ratkowsky, et al. (2019) was for *Hydrocotyle vulgaris*, a species that has oblate leaves and an MP value slightly smaller than *π*/4. It is not surprising that the estimates of MP for the three cicada species also fell within this range because their forewing shapes are similar to the shape of elliptical leaves. Schrader et al. (2021) analyzed 3125 leaves (from 144 families and 780 species and subspecies) with different shapes. They defined three specific hierarchical classification schemes and found a wider numerical range of MP values, from 0.39 to 0.79. The lower the complexity of the wing shape of insects, the narrower the numerical range of MP values. In other words, the numerical value of MP will be more accurate for quantification of wing shapes and their conspecific variation or variation across the closely related species. Although most insect wings have no teeth, dissections, or lobes (which many leaves have), the presence of scales or hairs also gives them a specific complexity, which might lead to a certain of variation in the MP value. Because insects’ wing size and shape are largely influenced by environmental factors (Dellicour et al., 2017), the variation in MP values can be indirectly used to reflect the influence of climate and other environmental conditions on different populations of the same species.

In previous studies on leaf shapes, the leaf roundness index (RI = 4*πA*/*P* ^2^) and leaf dissection index (DI = P/2$\sqrt{\pi A}$) (where *P* is the leaf perimeter and *A* is the leaf area) were frequently used to describe the complexity of leaf shapes (Kincaid & Schneider, 1983; Niinemets, 1998; Peppe et al., 2011; Santiago & Kim, 2009; Thomas & Bazzaz, 1996). Li, Quinn, Niinemets, et al. (2022) pointed out the shortcomings of RI and DI as parameters for description of leaf shapes, especially elliptical and oval leaf shapes that deviate significantly from a circular form. Indeed, these indices fail to reflect leaf shape. To describe the complexity of elliptical or oval‐shaped leaves, they developed the ellipticalness index (EI = 4*A*/π*LW*) based on the Montgomery equation. For insects, we can say that most insect wings are subelliptical when they are unfolded. Thus, MP values can also be used as an indicator to quantify the wing‐shape complexity of insects. Therefore, both MP values and the ellipticalness index appear to be valid measures of wing‐shape complexity.

### 4.3 Effects of sampling sites and time

We sampled *C*. *atrata* (Ca) at two sites during two periods to increase the sample size. Nevertheless, the distance between the two sample sites was only 13.7 km, and there is no evidence that either site had environmental differences large enough to alter the morphological features of those insects (Hou et al., 2018). It is apparent that the age of the Ca cicadas sampled in July was different from that of cicadas collected in June in the second round of sampling. However, age of individual insects appears to play a very minor role in affecting MP values within a species. Huang et al. (2021) calculated MP values of moso bamboo leaves of different culm ages, from one to seven years. Although MP values varied significantly with culm age, the numerical differences among MP values were so small that using the estimate of MP based on the pooled data for different culm ages caused only <4% mean absolute deviation in calculating leaf area of bamboo. Our fitted results confirmed this point, with the correlation coefficient of the Ca (*r* = 0.9762) being even slightly higher than that of the Pk (*r* = 0.9759), which was collected at only one site. In addition, there was no apparent separation in data points of ln *A* vs. ln *LW* around the regression line (Figure 3a).

In the present work, we confirmed the validity of the Montgomery equation (ME) in calculating the forewing area of three cicada species, and the estimated Montgomery parameters were approximately 0.7. The ME had a good goodness‐of‐fit for each species, and this exhibits that wing area can be approximately expressed as the product of wing length and width, multiplied by a correction factor 0.7. For the pooled dataset, the ME was still valid for calculating wing area, which suggests that this method can be potentially extended to other insect species with similar wing types.

## CONFLICT OF INTEREST

The authors declare no conflict of interest.

## AUTHOR CONTRIBUTIONS

**Kexin Yu:** Investigation (equal); Writing – original draft (equal). **Gadi V. P. Reddy:** Formal analysis (equal); Writing – review & editing (equal). **Julian Schrader:** Supervision (equal); Writing – review & editing (equal). **Xuchen Guo:** Investigation (equal). **Yirong Li:** Investigation (equal). **Yabing Jiao:** Investigation (equal). **Peijian Shi:** Formal analysis (equal); Investigation (equal); Supervision (equal); Writing – original draft (equal).

## Data Availability

All the raw data can be found in Dryad, a public repository (see Shi, 2022). https://doi.org/10.5061/dryad.4xgxd25bg.
